# Mast Cell-Intervertebral disc cell interactions regulate inflammation, catabolism and angiogenesis in Discogenic Back Pain

**DOI:** 10.1038/s41598-017-12666-z

**Published:** 2017-10-02

**Authors:** Matthew G. Wiet, Andrew Piscioneri, Safdar N. Khan, Megan N. Ballinger, Judith A. Hoyland, Devina Purmessur

**Affiliations:** 1 Department of Biomedical Engineering, The Ohio State University, Columbus Ohio, 201 Davis Heart and Lung Research Institute, 473 W 12th Avenue, Columbus, Ohio 43210 USA; 2 Department of Orthopedics, The Ohio State University Wexner Medical Center, 1070 OSU CarePoint East, 543 Taylor Avenue, Columbus, Ohio 43203 USA; 3 Department of Internal Medicine, Division of Pulmonary, Critical Care and Sleep Medicine, The Ohio State University, 201 Davis Heart and Lung Research Institute, 473 West 12th Avenue, Columbus, Ohio 43210 USA; 4 Division of Cell Matrix Biology and Regenerative Medicine, School of Biological Sciences, Faculty of Biology, Medicine and Health, The University of Manchester, Stopford Building, Oxford Road, Manchester, M13 9PT United Kingdom; 5 NIHR Manchester Musculoskeletal Biomedical Research Centre, Manchester Academic Health Science Centre, Central Manchester NHS Foundation Trust, Manchester, United Kingdom

## Abstract

Low back pain (LBP) is a widespread debilitating disorder of significant socio-economic importance and intervertebral disc (IVD) degeneration has been implicated in its pathogenesis. Despite its high prevalence the underlying causes of LBP and IVD degeneration are not well understood. Recent work in musculoskeletal degenerative diseases such as osteoarthritis have revealed a critical role for immune cells, specifically mast cells in their pathophysiology, eluding to a potential role for these cells in the pathogenesis of IVD degeneration. This study sought to characterize the presence and role of mast cells within the IVD, specifically, mast cell-IVD cell interactions using immunohistochemistry and 3D *in-vitro* cell culture methods. Mast cells were upregulated in painful human IVD tissue and induced an inflammatory, catabolic and pro-angiogenic phenotype in bovine nucleus pulposus and cartilage endplate cells at the gene level. Healthy bovine annulus fibrosus cells, however, demonstrated a protective role against key inflammatory (IL-1β and TNFα) and pro-angiogenic (VEGFA) genes expressed by mast cells, and mitigated neo-angiogenesis formation *in vitro*. In conclusion, mast cells can infiltrate and elicit a degenerate phenotype in IVD cells, enhancing key disease processes that characterize the degenerate IVD, making them a potential therapeutic target for LBP.

## Introduction

Low back pain (LBP) is a debilitating disorder that affects nearly 80% of the population at least once in their lifetime, costing more than $100 billion in lost wages, decreased productivity, and medical costs in the US alone^1^. One of the leading sources of LBP is intervertebral disc (IVD) degeneration^2^, which occurs as a result of the complex interplay between biological and biomechanical etiology of the spine leading to general tissue degradation and ultimately loss of function of the IVD^3,4^. Under normal conditions, the IVD is avascular and aneural, consisting of three integrated structures: the inner gelatinous nucleus pulposus (NP), the outer lamellar annulus fibrosus (AF), and the cartilaginous endplates (EP) connecting the IVD to the vertebral bodies^5^. The avascular nature of the healthy IVD, creates a poor microenvironment for tissue healing and repair. In disease, degeneration of the IVD is often associated with inflammation, immune cell infiltration and neo-vascularization – processes that occur during normal tissue healing; however in the hostile microenvironment of the IVD these processes augment catabolism and pain rather than instigating repair^6^. While inflammation in the degenerate IVD has been well characterized and neo-vascularization to a lesser extent^7–11^, the specific role that immune cell infiltration plays in disease pathogenesis is not well understood especially as the healthy IVD is largely immune-privileged. The immune cells’ response to acute tissue injury and role in inflammation suggests that they could potentially play a critical role in promoting catabolic remodeling within the IVD, leading to enhancement of neo-angiogenesis and pain.

The cells within the healthy IVD, in particular the NP, function to maintain a constant balance between catabolic and anabolic remodeling of the extracellular matrix (ECM), which is primarily composed of proteoglycans and collagens I/II. Common anabolic factors that promote matrix biosynthesis include insulin-like growth factor (IGF), transforming growth factor-β (TGF-β), and bone morphogenic proteins (BMPs)^12^. Catabolic factors that enhance matrix degradation include matrix metalloproteinase (MMPs), members of a disintegrin and metalloproteinase with thrombospondin motifs (ADAMTS), and aggrecanases. In disease, there is an upregulation of pro-inflammatory cytokines IL-6, IL-1β, and TNF-α which influence catabolic factors, perpetuating degradation of disc tissue^13–15^. Inflammatory cytokines can come from both the IVD cells^16^ or from infiltrating immune cells^17^. Overall this microenvironment leads to a decrease in ECM biosynthesis, particularly proteoglycans, leading to decreased water content, reduced production of anabolic factors and increased catabolism.

Previous studies have demonstrated that with degeneration there is also an upregulation of VEGF and NGF, that can promote neurovascular ingrowth^18–20^. Angiogenesis has been known to contribute to many different pathological conditions including tumor progression, osteoarthritis^21^ and rheumatoid arthritis^22,23^ and plays a key role in recruiting nerves to innervate various regions^24,25^. While the healthy intervertebral disc is avascular and aneural, it is thought that degeneration induces structural and biochemical changes that contribute to angiogenesis and subsequent innervation of the disc, effectively sensitizing the IVD and resulting in low back pain^26^. This process is likely further perpetuated by the activity of immune cells that have infiltrated the disc.

In the healthy IVD, immune cell access to the IVD is restricted by a physical barrier and molecular mechanisms^27^. However, changes in the IVD’s structure and cellular phenotype with degeneration result in this barrier being compromised in disease, allowing the infiltration of immune cells. Previous work has demonstrated the importance of macrophages and the ability of the NP cells to produce factors that serve as chemo-attractants for macrophages^28^. Currently, macrophages are thought to play a role predominantly in herniations of the disc through resorption of the extracellular matrix components of the IVD^29,30^. T lymphocytes have also shown to be present in degenerative discs^31^, causing an upregulation of IL-17^32^ and production of other important cytokines in IVD degeneration^33^; whereas the role of innate immune cells, i.e. mast cells is less well defined.

Mast cells are hematopoietic cells that originate from bone marrow pluripotent precursors^34^. Mast cell progenitors are found in the blood until they enter their target tissue and differentiate into mature mast cells via differentiation factors like IL-3 or stem cell factor (SCF). Mast cells are the “first responders” during tissue injury, releasing pre-formed granules during degranulation that contain cytokines, growth factors, and enzymes (e.g. TNF-α, IL-6, NGF, VEGF, substance P, ADAMTS5 and Tryptase) and are specifically recruited to tissues to augment angiogenesis, healing and repair^35^. These preformed granules are released during activation by IgE, synthetic compounds, and mechanical stimulation^34,36^. In an experimental model of arthritis utilizing glucose-6-phosphate isomerase inhibition, it was shown that mast cell knockout mice were protected against joint destruction and angiogenesis via αvβ3 integrin activation^37^. Chondrocytes co-cultured with activated mast cells promote degradation of proteoglycans^38^, and may contribute to structural degradation and degeneration of the IVD in a similar fashion. Additionally, mast cells are responsible for recruitment of other cell types (e.g., macrophages) that promote matrix remodeling in rheumatoid arthritis, via the release of chemokines such as chemokine (C-C motif) ligand 2 (CCL2/MCP-1)^39^. Mast cells are also present and active in a number of chronic pain conditions which include migraines, irritable bowel syndrome, fibromyalgia, as well as rheumatoid and osteoarthritis^40–43^. The effect of mast cells is not only limited to degranulation, as they synthesize cytokines and chemokines de novo, which perpetuate the inflammatory conditions. As LBP is considered a chronic pain state, it is reasonable to assume that mast cells may play an integral role in degeneration and sensitization of the IVD leading to discogenic pain. In the current study, we sought to understand the potential role that mast cells play in degeneration and pain mechanisms within the diseased IVD.

Our central hypothesis is that mast cells are upregulated in the painful IVD and that upon activation they augment catabolic, inflammatory and angiogenic processes as well as initiate further immune cell recruitment perpetuating a chronic inflammatory painful IVD microenvironment. The aims of this study were to: (1) confirm and quantify the presence of mast cells in painful human IVD tissue as well as examine a potential mechanism for recruitment by chemo-attractants SCF and CCL2/MCP-1, and (2) investigate the effects of soluble factors from mast cells on inflammatory, angiogenic and catabolic gene expression in IVD cells and conversely how soluble factors from healthy and degenerate IVD cells influence mast cells.

## Results

### Mast cell Localization in the human Intervertebral Disc

**Figure 1:**
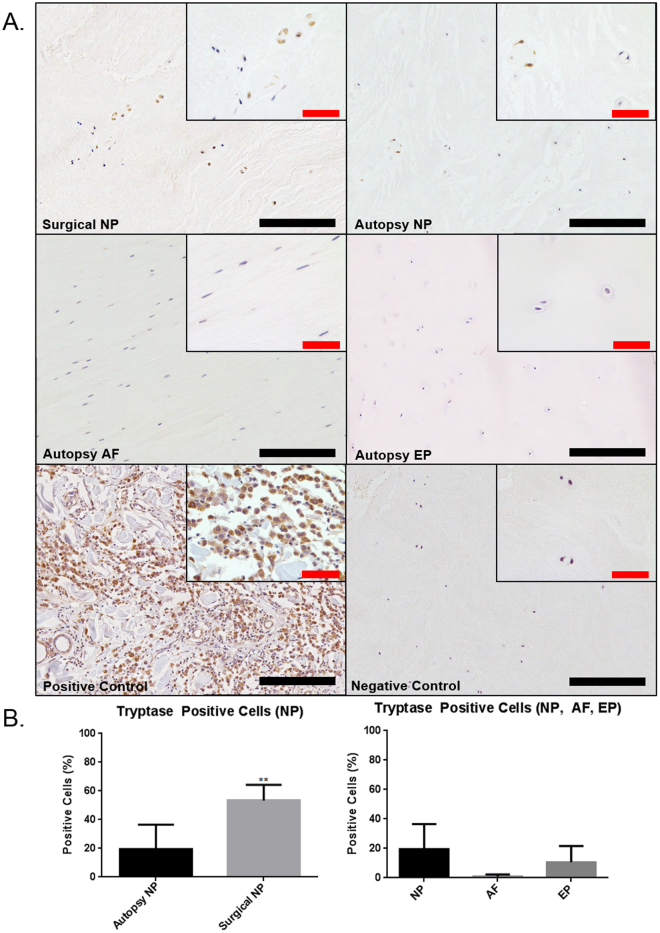
Immunohistochemical staining for mast cell specific tryptase in human IVD tissue (**A**). The positive control was tissue taken from a human mast cell tumor. Quantification of percent positive cells for tryptase demonstrated significant upregulation of tryptase in human surgical tissue samples compared to autopsy controls (**B**) (p = 0.0047) (Autopsy N = 7, Surgical N = 6). Black scale bar = 200 µm, Red scale bar = 50 µm.

IHC staining was used to investigate the presence of mast cell specific phenotypic marker tryptase within the human IVD. Tryptase staining was demonstrated in both painful human surgical IVD specimens and cadaveric IVD tissue obtained at autopsy (Fig. 1A). When comparing autopsy NP to painful surgical NP tissue there was a significantly higher percentage of tryptase positive cells in the painful surgical discs (53.3%) when compared to non-painful autopsy samples (19.3%) (p = 0.0047) (Fig. 1B). Positive staining was seen in all regions, however regional differences of mast cell tryptase points towards a trend of a higher presence of mast cells in the NP/EP than in the AF (Fig. 1B), although this was not statistically significant.

### Recruitment of mast cells into the Intervertebral Disc

**Figure 2:**
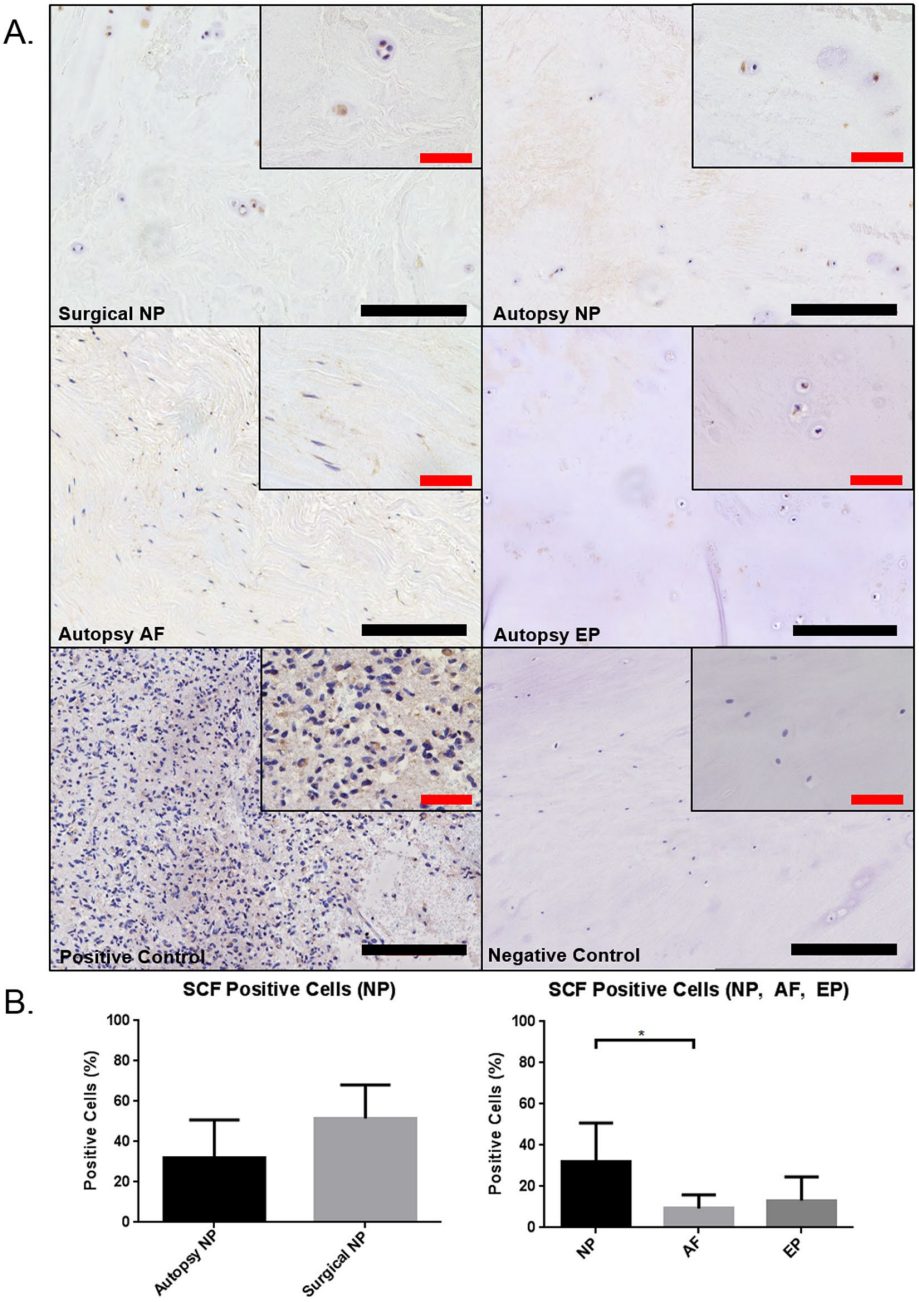
Immunohistochemical staining for mast cell chemoattractant stem cell factor (SCF) in human IVD (SCF) (**A**). The positive control was tissue taken from human brain tissue. Quantification of percent positive cells for SCF demonstrated no significant differences between surgical and autopsy specimens (p = 0.081) however regional differences were observed between NP and AF (**B**) (p = 0.017) (Autopsy N = 7, Surgical N = 6). Black scale bar = 200 µm, Red scale bar = 50 µm.

**Figure 3:**
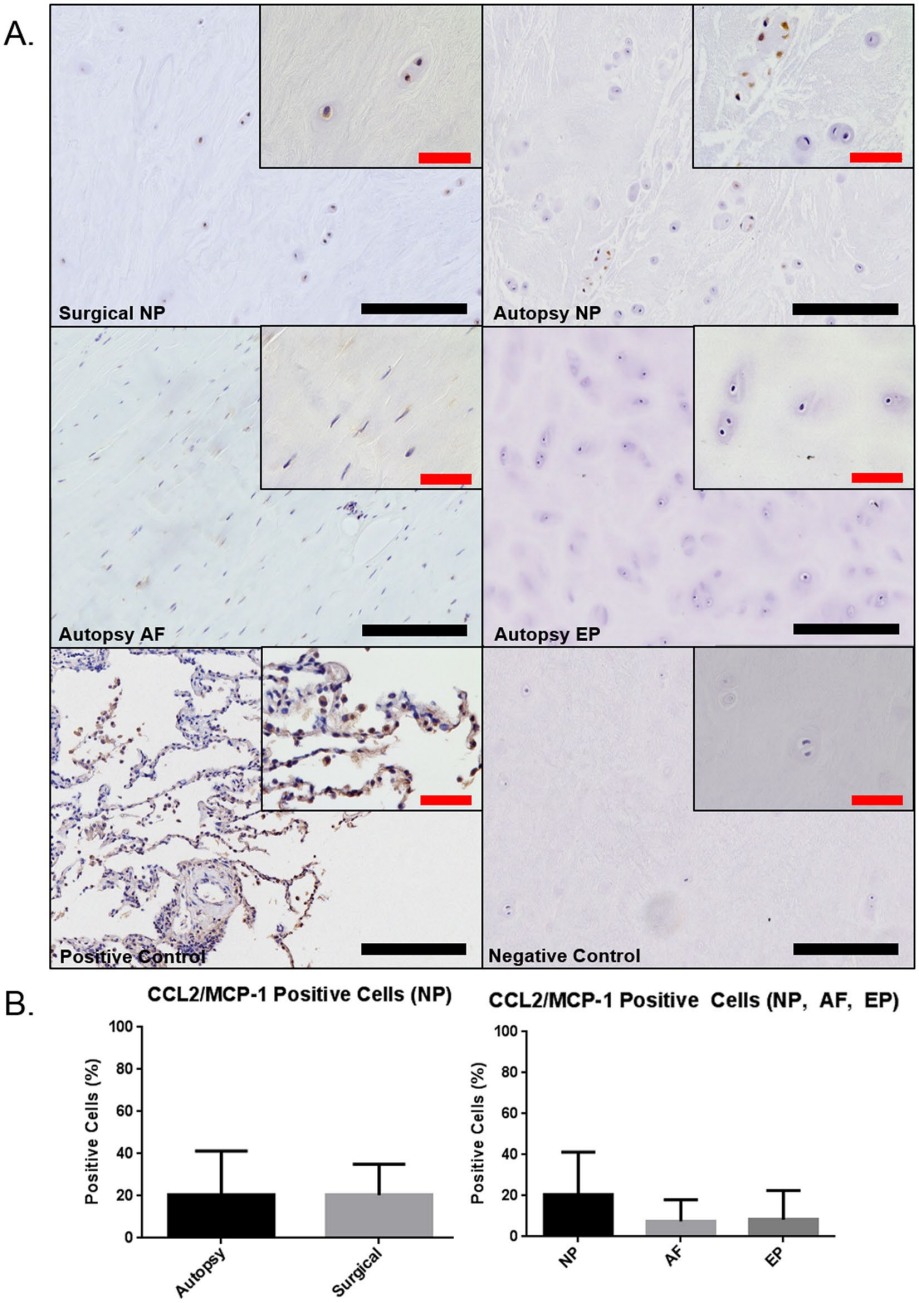
Immunohistochemical staining for immune cell chemoattractant CCL2/MCP-1 (**A**). The positive control was taken from human lung tissue. Quantification of percent positive cells for CCL2/MCP-1 demonstrated no significant differences between surgical and autopsy (p = 0.17) or between regions (p = 0.87) (**B**) (Autopsy N = 7, Surgical N = 6). Black scale bar = 200 µm, Red scale bar = 50 µm.

CCL2/MCP-1 and SCF are known chemo-attractants for innate immune cells such as mast cells and macrophages. IHC and the percentage of tryptase positive cells were used to quantify the presence of SCF and CCL2/MCP-1 in the painful surgical and cadaveric human IVD samples (Fig. 2A). SCF staining in autopsy samples revealed a significant increase in expression within the NP (32.1%) region when compared to the AF region (9.3%) (p = 0.017) (Fig. 2B). Painful, surgical NP showed a trend of increased SCF expression compared to autopsy NP (p = 0.081). As this is the first study to demonstrate SCF protein expression via IHC in the human IVD, we also confirmed expression at the gene level in all regions of the human IVD (Supplemental Figure). In contrast, CCL2/MCP-1 showed no significant differences in regional expression of autopsy IVD tissue (p = 0.17) or autopsy NP versus surgical NP (p = 0.87) (Fig. 3A & B).

### The effect of Mast Cell Conditioned Media (MCCM) on IVD Cells

**Figure 4:**
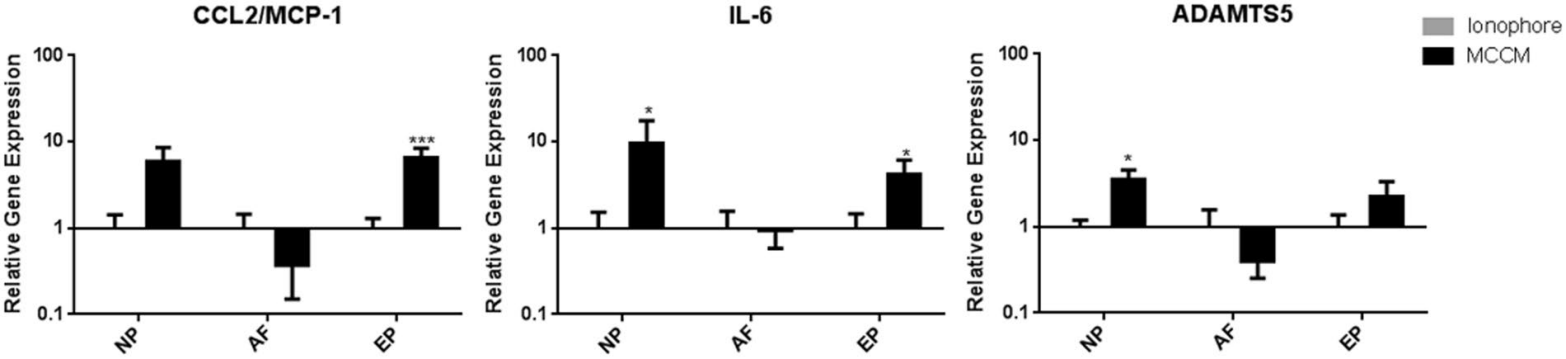
Gene expression for CCL2/MCP-1, IL-6, ADAMTS5 in bovine disc cells in response to mast cell conditioned media (MCCM). Significant upregulation of ADAMTS5 (p = 0.023), and IL-6 (p = 0.023) in NP as well as upregulation of IL-6 (p = 0.0244) and MCP-1 (p = 0.0003) in CEP relative to the ionophore control (N = 11 for NP/AF and N = 10 for EP).

To examine the effect of soluble factors secreted from mast cells on bovine IVD cells, IVD cells (NP, AF, EP) were treated with MCCM for 24 hours. Cell viability (Calcein/Ethidium) and proliferation (3-(4,5-dimethylthiazol-2-yl)-2,5-diphenyltetrazolium bromide (MTT)) assays demonstrated no significant differences between IVD cells exposed to MCCM compared to basal controls, respectively (Supplemental Figure). Inflammatory and pro-angiogenic factors, IL-6, ADAMTS5, and CCL2/MCP-1 were evaluated using qRT-PCR; mast cells significantly increased the expression of IL-6 (p = 0.023) and ADAMTS5 (p = 0.023) in NP cells compared to controls (Fig. 4). For EP cells, exposure to MCCM significantly increased CCL2/MCP-1 (p = 0.0003), as well as IL-6 expression (p = 0.0244). Notably, there were no differences in gene expression for the AF (Fig. 4).

### The effect of Disc Cell Conditioned Media (DCCM) on Mast Cells

**Figure 5:**
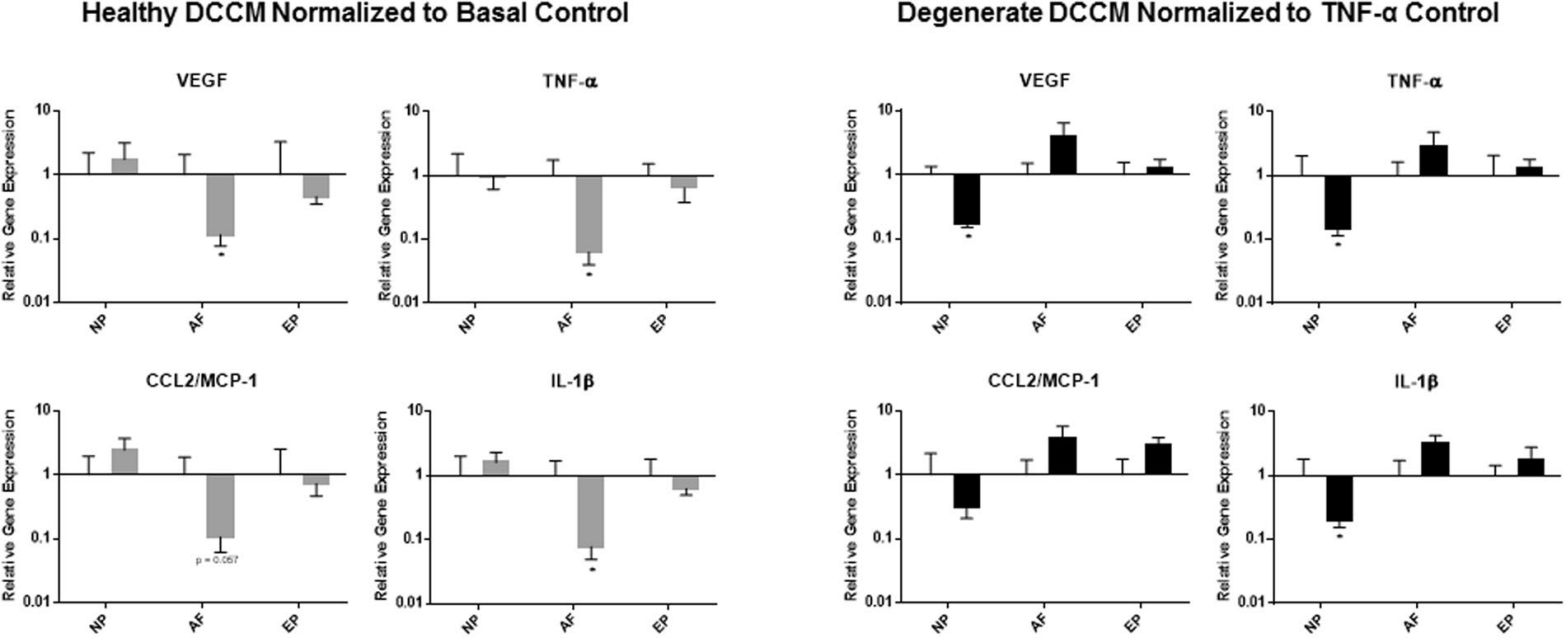
Gene expression of mast cells in response to disc cell conditioned media (DCCM). Significant decreases in mast cell expression of key inflammatory/angiogenic markers (VEGF, TNF-α, and IL-1β) (p = 0.015, p = 0.013, p = 0.013) when exposed to healthy AF DCCM relative to mast cells cultured in basal control conditions. Degenerate NP DCCM also decreased expression of TNF-α (p = 0.049) in mast cells relative to mast cells cultured with TNF-α (TNF-α control) (N = 4).

**Figure 6:**
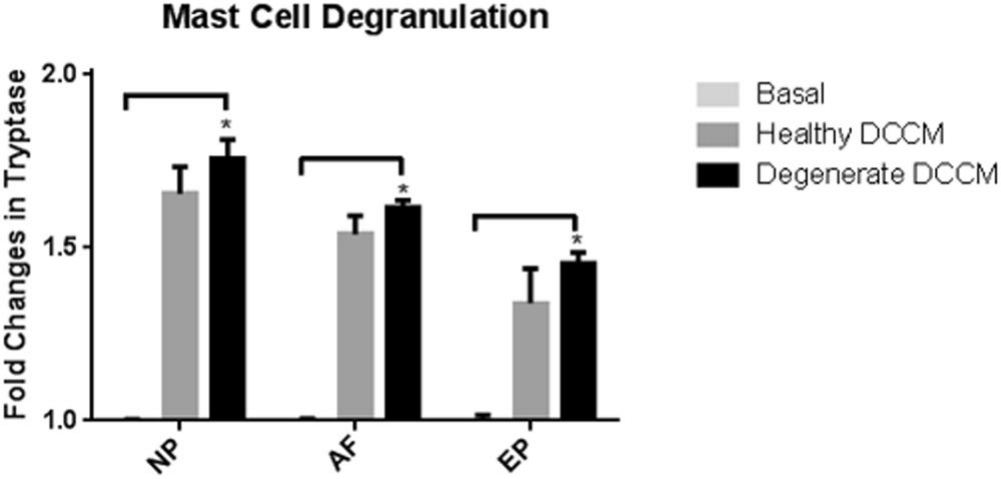
Mast cell activation as measured by the mast cell degranulation assay and release of tryptase into the media upon exposure to IVD DCCM. All three regions (NP, AF EP) of the degenerate IVD increased degranulation of mast cells relative to basal control conditions (p = 0.011, p = 0.0048, p = 0.0048 respectively). Healthy DCCM had a trend of increased degranulation (N = 4).

Heathy and degenerate DCCM from all IVD cell types was used to determine the effects of IVD cells on mast cells. After a 24 hour treatment, significant decreases were observed in the expression of VEGF (p = 0.0286), TNF-α (p = 0.0286) and IL-1β (p = 0.0286) and a trend of decreased expression in CCL2/MCP-1 (p = 0.057) in mast cells treated with healthy AF DCCM compared to untreated basal controls and degenerate AF DCCM, suggesting an inhibitory role for AF cells on mast cell phenotype (Fig. 5). Interestingly, degenerate NP DCCM significantly decreased VEGF (p = 0.0286), TNF-α (p = 0.0286), and IL-1β (p = 0.0286) expression. Neither healthy nor degenerate EP DCCM altered mast cell gene expression of the chosen inflammatory/angiogenic markers (Fig. 5). Mast cell activation/degranulation was evaluated in the presence of healthy and degenerate DCCM from all IVD cell types. A significant increase in mast cell degranulation with degenerate DCCM compared to basal control was noted for all IVD cell groups (NP, AF, EP) (p = 0.011, p = 0.0048, p = 0.0048 respectively) (Fig. 6). There were no significant differences in mast cell degranulation between healthy DCCM and basal control.

### The effect of Disc-Mast cell interactions on Angiogenesis

**Figure 7:**
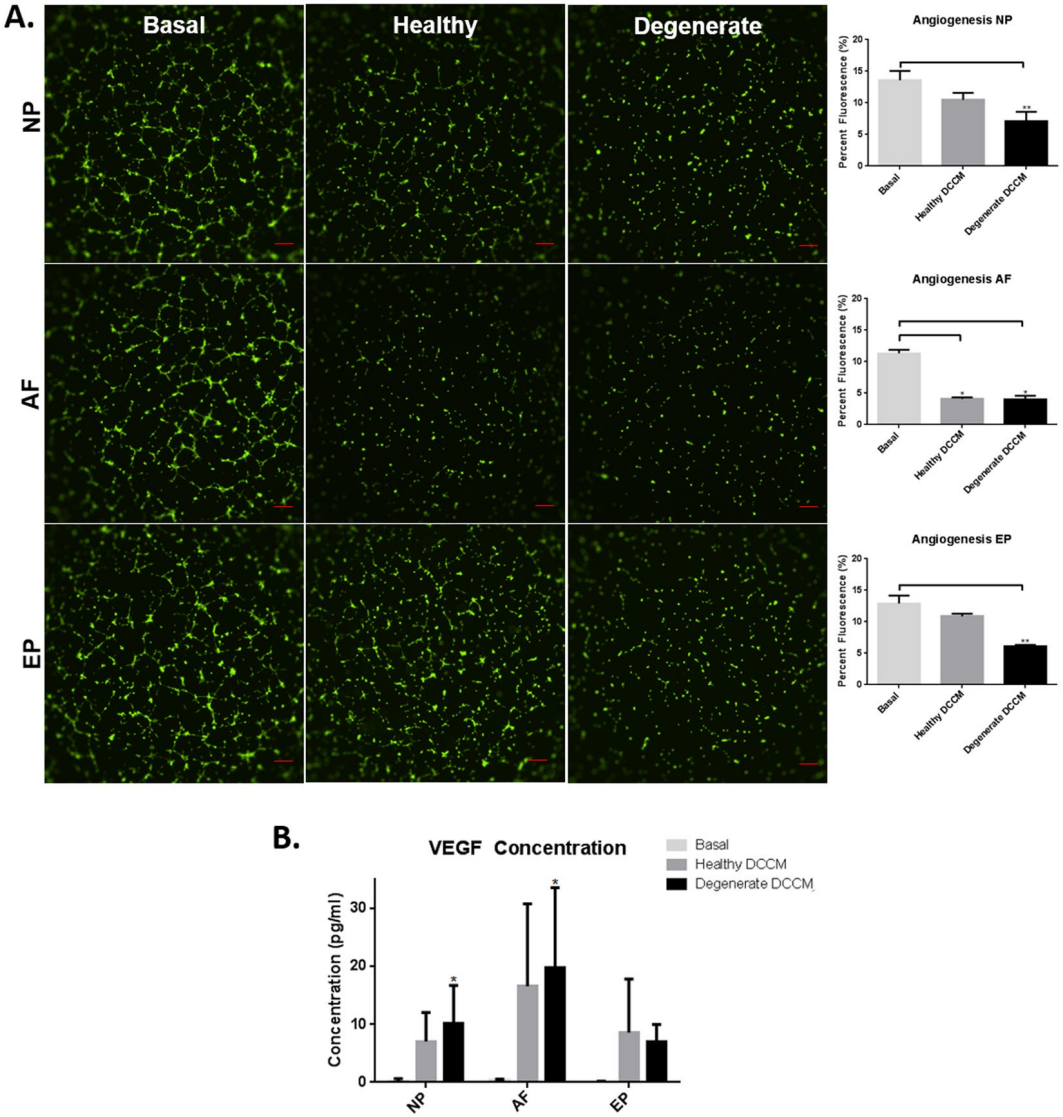
(**A**) Endothelial tubular formation in the presence of media from mast cells cultured in the IVD DCCM. Healthy and degenerate AF DCCM (p = 0.015), degenerate NP DCCM (p = 0.0002), and degenerate EP DCCM (p = 0.0002) all significantly down regulated endothelial tubular formation (N = 4). (**B**) Significant upregulation of VEGF secretion in mast cells exposed to AF DCCM and NP DCCM was observed compared to basal controls (p = 0.0066 and p = 0.046 respectively) (N = 3).

To examine the functional effects of DCCM-mast cell interactions on angiogenesis, DCCM from mast cell cultures was added to HUVECs, and tubular formation analyzed. Results revealed a significant decrease in endothelial tubular formation when HUVECs were treated with healthy and degenerate AF DCCM compared to basal control, suggesting that AF cells can inhibit mast cell induced angiogenesis (p = 0.015) (Fig. 7A). NP and EP DCCM both demonstrated a significant decrease in endothelial tubular formation in degenerate conditions compared to basal control, and no difference in tubular formation with healthy DCCM (p = 0.0002, p = 0.0002 respectively). Protein analysis of VEGFA expression in mast cells treated with DCCM showed that degenerate AF DCCM and NP DCCM groups significantly increased VEGFA expression in mast cells compared to basal control media (p = 0.0066, p = 0.046 respectively) (Fig. 7B).

## Discussion

Immune cells such as mast cells play a critical role in the pathogenesis of degenerative musculoskeletal diseases such as rheumatoid arthritis and osteoarthritis as well as chronic pain conditions such as irritable bowel syndrome, migraines and fibromyalgia^44–47^, yet their role within the painful degenerate/diseased IVD is not well understood. A limited number of studies have identified mast cells in the IVD and speculated as to the role they may play in LBP^48,49^, however the current study is the first to examine and characterize disc cell-mast cell interactions and their potential role in neo-angiogenesis with respect to LBP. Specifically, mast cells were upregulated in painful human surgical NP IVD tissue compared to cadaveric controls. Clinically, this suggests that they play a significant role in disease pathogenesis especially as they secrete a number of pro-inflammatory, neurovascular and catabolic factors^34^ that help to create a permissive microenvironment for neurovascular ingrowth and pain. Disc cell-mast cell interactions support this concept with both mast cells and degenerate disc cells inducing a pro-inflammatory/catabolic/angiogenic phenotype in NP and EP cells or mast cells respectively. Interestingly, healthy AF cell-mast cell interactions inhibited the pro-inflammatory/catabolic/angiogenic response elicited by mast cells.

The IVD is immune-privileged in its healthy state therefore potential factors that may be involved in mast cell recruitment such as SCF and CCL2/MCP-1 were investigated^50–52^. In this study, tissue from both surgical and autopsy IVDs expressed SCF and CCL2/MCP-1, with regional differences and enhanced expression of SCF in the NP compared to AF. SCF and CCL2/MCP-1 were chosen due to their role in mast cell recruitment during tissue injury and repair^53,54^ and their recent identification as biomarkers in patients with LBP^55,56^. The regional differences in SCF expression indicate that mast cell recruitment to the center of the IVD is due to higher SCF expression from the NP cells. Lesions in the EP could expose the IVD to a higher concentration of hematopoietic precursor cells from the bone marrow and increased expression of SCF in the NP would provide a chemotactic path for immune cells into the IVD. Furthermore, SCF induces neuronal outgrowth from c-kit positive dorsal root ganglion cells^57^ and could play a role in facilitating nerve ingrowth and nociception in the IVD.

Once the mast cells have infiltrated the disc, there must be a sufficient stimulus to activate them to release their granules into the immediate environment. Degranulation can be caused by a variety of different natural or synthetic materials (IgE, Compound 48/80, A23187, etc.)^58,59^. Our study demonstrated that DCCM induces degranulation of mast cells suggesting that degenerate IVD cells secrete soluble factors that activate mast cells. Indeed SCF activates mast cells and may play a role in mast cell degranulation in the IVD, promoting inflammation, angiogenesis and pain^60,61^. As IVD degeneration often leads to altered biomechanics and increased loads experienced by the cells within it, it should be noted that mast cells can also be activated under mechanical loads up to 10% strains via an RGD-integrin pathway^36^. There is likely a combination of mechanical and biochemical cues that contribute to activation, but these mechanisms are beyond the scope of the current study. Here we demonstrated that mast cell activation induces a pro-catabolic/inflammatory phenotype in NP and EP regions of the IVD as shown by increases in ADAMTS5, IL-6, and CCL2/MCP-1 expression at the transcriptional level, key factors involved in degeneration of the IVD^62^. Degeneration is likely further amplified by the mast cells themselves secreting bioactive ligands such as TNF-α, VEGF, TGFβ, IL-6, MMP9, and ADAMTS5 into the local environment^34^. This creates a permissive microenvironment ideal for neo-angiogenesis and neo-innervation to occur, and helps to perpetuate painful mechanisms in the diseased IVD^6,19,63,64^. Interestingly, AF cells did not respond to mast cell soluble factors as the NP and EP cells suggesting that mast cells were unable to alter the AF cell phenotype.

This study also demonstrated that the IVD cells can have a profound effect on the function of mast cells *in vitro*. Soluble factors from EP cells demonstrated limited effect on mast cells, while healthy AF and degenerate NP cells induced significant down regulation of inflammatory and angiogenic factors TNF-α, IL-1β, and VEGF with a trend in CCL2/MCP-1 in mast cells. This suggests an inhibitory/regulatory role for healthy AF and degenerate NP cells in immune-IVD cell interactions. This difference can be explained through the native structure of the IVD wherein the AF is constantly exposed to the outer systemic environment and thus must constitutively produce factors to limit angiogenesis/inflammation. On the other hand, the NP is protected within the disc and has an acute response to the infiltration of mast cells by attempting to suppress inflammation. Mast cells contribute to a number of angiogenic processes *in vivo* ^65–68^ therefore the effect of disc cells on the ability of mast cell to promote angiogenesis was assessed. Mast cells, when exposed to degenerate DCCM from AF and NP cells, demonstrated an increased VEGFA protein expression however the EP had no effect. This was further evaluated by examining the effect of IVD cells on mast cells’ ability to induce endothelial tubular formation using a tubular assay. While VEGF expression was increased in NP and AF DCCM-mast cell cultures, healthy and degenerate AF cells actually decreased tubular formation with degenerate NP/EP cells also decreasing tubular formation to a lesser extent. IVD cells are also known to secrete anti-angiogenic factors such as noggin^64^ as well as chondroitin sulfated proteoglycans^7^ which may influence the inhibitory effects observed here, antagonizing VEGFA and require further investigation. The dominant effects observed particularly in AF-mast cells cultures may be explained by the fact that since the AF is in constant contact with the external environment, it functions as a barrier to the systemic immune system and down-regulates pro-inflammatory and angiogenic processes, yet the NP and EP are effectively hidden from the immune system in the healthy state and only produce these “factors” as a defense mechanism in response to acute injury or degeneration.

**Figure 8:**
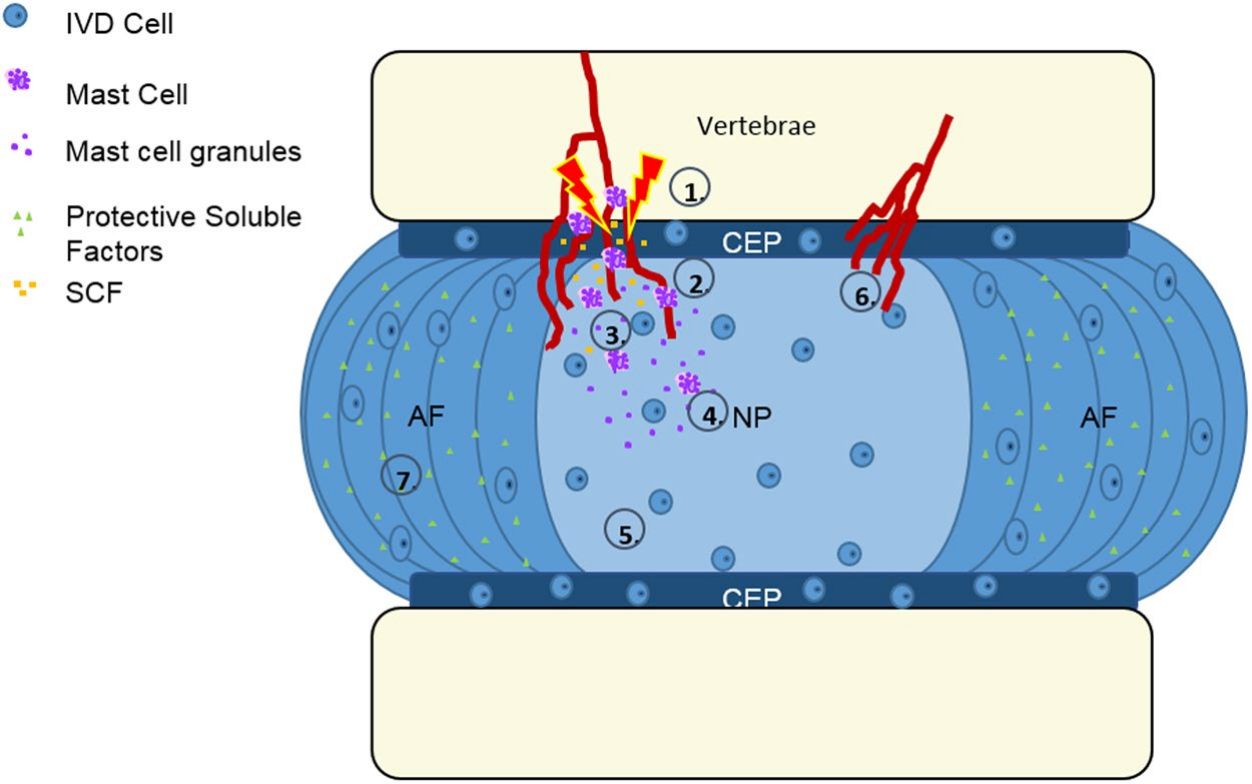
Hypothetical model wherein the disc undergoes injury/degeneration (1), followed by mast cell recruitment by SCF (2 & 3). Mast cells are then activated (4) in the degenerate environment and elicit catabolic changes in the IVD microenvironment (5) promoting neurovascular ingrowth and pain (6). In the healthy IVD, the AF produces protective soluble factors that help to maintain an immune-privileged microenvironment, inhibiting mast cell activation and angiogenesis (7).

In Fig. 8, a hypothetical model of IVD-mast cell interactions in the context of IVD degeneration is presented. In the healthy state, the disc, in particular the AF, acts as both a physical and biochemical barrier to inflammation and immune cell infiltration. During aging and degeneration, injury and modic changes in the vertebral endplate creates bone marrow lesions that expose the IVD and EP to the bone marrow and a rich supply of immune cell progenitors. Once exposed, recruitment of mast cells into the IVD is accomplished through up-regulation of the mast cell chemoattractant SCF. Mast cells can then be activated through several different mechanisms, one of them likely being cellular interactions with IVD cells. Upon degranulation, inflammatory cytokines are released into the immediate microenvironment, inducing a catabolic/pro-inflammatory phenotype in the disc cells which then secrete factors that further promote mast cell activation. This degenerative cycle produces an environment rich in angiogenic and neurogenic factors that can recruit local blood vessels and neurites into the disc resulting in a state of chronic inflammation and pain.

## Conclusion

To our knowledge, this is the first study to explicitly explore the presence and function of mast cells in the intervertebral disc. Here we demonstrated that mast cells are present in the painful IVD and that they likely play a key role in degeneration of the IVD. We provide evidence that mast cells are able to i) stimulate an inflammatory and catabolic phenotype in IVD cells specifically NP and EP cells, and vice versa, and ii) that healthy AF cells can inhibit activation of mast cells and a pro-angiogenic phenotype in these cells. Future studies will be aimed at identifying specific factors secreted by the mast cells or IVD cells that may function as potential targets for treatment of discogenic back pain.

## Materials and Methods

All reagents were obtained from Sigma Aldrich/Fisher Thermo-Scientific unless otherwise stated. Materials, data and associated protocols are available upon request to the corresponding author.

### Cell culture

#### IVD and EP Cell Culture

IVDs were obtained from eleven skeletally mature bovine (13–15 months) tails (n = 11) and NP, AF and (N = 10) EP tissues dissected. Cells were isolated using a protease enzyme from strep *Streptomyces griseus* (0.05 g/5 ml) in digestion media (DMEM, 1% penicillin/streptomycin (P/S), 0.5% Fungizone) for 1 hour, followed by collagenase I (AF) or collagenase II (NP/EP) digestion for 12 hours (0.003 g/25 ml) as previously described^69^. Cells were expanded in disc cell media (DMEM, 10% FBS, 1% P/S, 50 μg/ml ascorbic acid, 4.5 g/ml glucose) in standard culture conditions (5% CO_2_, 37 °C), and fed every 3 days until confluent. Cells were used at a passage <P3. One limitation of the study is the potential lack of cross-species reactivity between the bovine IVD cells and human mast cells; however to reduce variability and use a consistent cell source we chose bovine because of its mature cell phenotype similar to the human IVD cells^70^.

#### Mast cell culture

**Figure 9:**
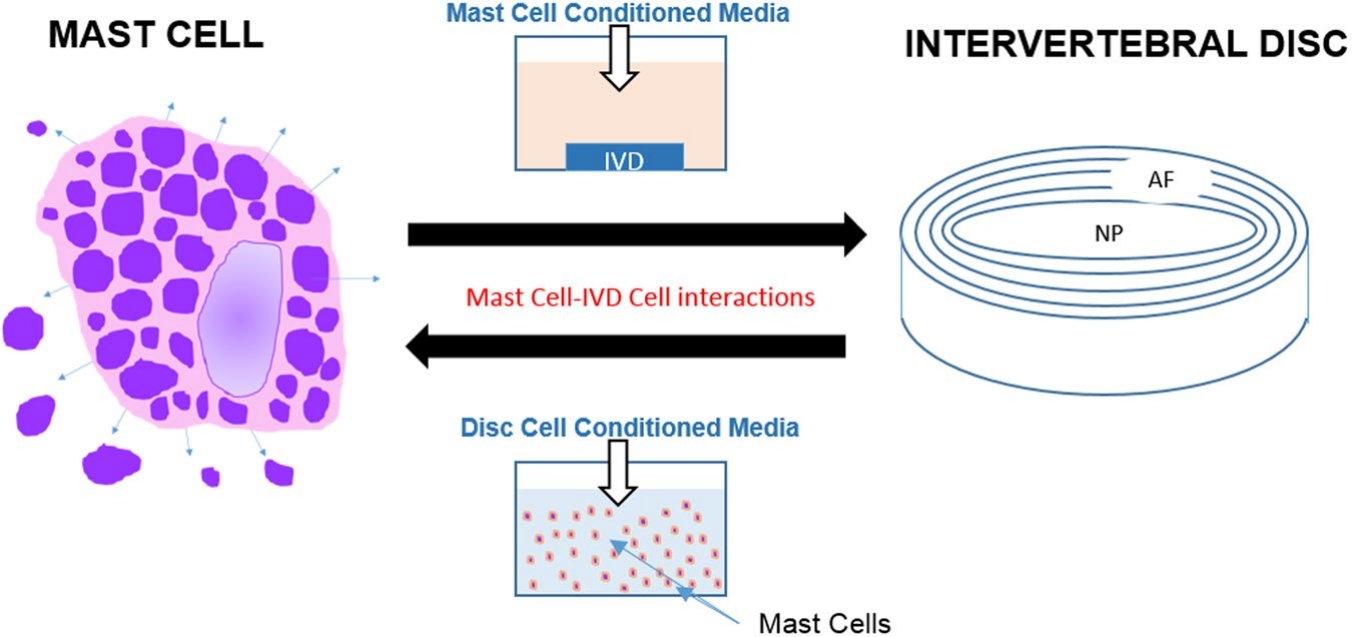
Experimental model to characterize the effect of mast cells in the IVD microenvironment. Conditioned media from activated mast cells was applied to 3D *in vitro* constructs containing NP, AF, or EP cells. Healthy and degenerate IVD cell conditioned media (NP, AF, EP) was applied to mast cells in suspension.

The HMC-1 leukemic mast cell line was provided as a generous gift from Dr. J.H. Butterfield (Mayo Clinic), and will be referred to as mast cells in this article. They were expanded in Iscove’s Modified Dulbecco’s Medium (IMDM) supplemented with 10% FBS, 1% P/S, and 1.2 mM α-Thioglycerol at (5% CO_2_, 37 °C) at a density of 5.0 × 10^5^–9.0 × 10^5^ cells/ml until ready for experimentation. Figure 9 describes the in-vitro cell culture techniques used for these studies. The HMC-1 cell line is a well characterized and thoroughly validated human mast cell line considered very similar to in vivo mast cells^71^; however as this is an immortalized cell it does have a c-kit mutation that allows for easier handling and survivability. It has been shown that these cells may have reduced tryptase levels when compared to mature human skin mast cells^72^.

### Mast Cell Localization in the Intervertebral Disc

#### Immunohistochemistry for Mast Cell Tryptase

Human IVD tissue specimens (surgical) were obtained and approved by informed consent from all participants in accordance with relevant The Ohio State University (Columbus, Ohio) Institutional Review Board (IRB) guidelines and regulations under IRB# 2105H0385. All experimental protocols for human surgical tissue specimens were approved by The Ohio State University IRB. Tissue was procured from patients with LBP (19–63 years) undergoing micro-discectomy, laminectomy, spinal decompression, or spinal fusions. Table 1 lists the sex, age and level of IVDs used for immunohistochemical analysis. Surgical specimens were fixed in neutral buffered formalin and processed in paraffin for histological assessment. Human IVD tissue specimens, (43–71 years), obtained from cadaveric spines within 36 hours of death (Co-operative Human Tissue Network, Columbus Ohio) were isolated and processed as for surgical specimens described above. Immunohistochemistry (IHC) was performed using mast cell marker tryptase (1:200 Abcam ab2378). Briefly, tissue slides were deparaffinized, rehydrated, blocked for endogenous peroxidase activity (0.3% H_2_O_2_ in MeOH), and antigens retrieved using a citrate buffer (90 °C, pH 6.0) for 20 minutes. Blocking for non-specific binding used 5% goat-serum (1% BSA-PBS, 5% goat serum, 0.05% tween, and 0.05% sodium azide). Primary antibodies were incubated for 18 hours in reducing antibody diluent (Dako) followed by incubation with the secondary antibody: biotinylated goat anti-mouse or biotinylated goat anti-rabbit (1:200, VectorLabs). Finally tissue sections were incubated with streptavidin-horse radish peroxidase and developed with 3,3-diaminobenzidine for 90 seconds (VectorLabs). Tissue (nuclei) was counterstained with Mayer’s or Gills No. 2 Hematoxylin (Electron Microscopy Sciences) and slides dehydrated and mounted. Positive controls were: human mast cell tumor (Tryptase), human brain (SCF), human lung (CCL2/MCP-1). Negative tissue type controls were performed without the use of primary antibody in order to exclude the possibility of false positives due to background staining. All antibody concentrations and DAB exposure times were kept the same across all human disc tissue specimens assessed for each specific protein (tryptase, SCF and CCL2/MCP-1) assessed. All images were captured on a Nikon TiE Inverted Microscope with a high resolution DS-U3 camera at 20x or 40x magnifications.

**Table 1:** Human autopsy and surgical specimen demographic information and level of surgery.

Autopsy				Surgical			
ID	Sex	Age	Level	ID	Sex	Age	Level
Hu1	Male	71	L4-L5	Hs2	Male	26	L5-S1
Hu2	Female	43	L4-L5	Hs7	Male	65	L3-L4
Hu3	Male	65	L4-L5	Hs10	Male	44	L5-S1
Hu4	Female	49	L4-L5	Hs11	Male	28	L5-S1
Hu5	Male	55	L4-L5	Hs12	Female	19	L4-L5
Hu6	Male	45	L4-L5	Hs16	Female	59	L5-S1
Hu7	Female	56	L4-L5	Hs17	Female	40	L5-S1
Hu8	Male	52	L4-L5				

#### Quantification of Immunohistochemical Staining

Sixteen representative images at 20x magnification were taken and stitched together at two different locations for each region of the autopsy IVD tissue sample (N = 7). Four images per surgical sample (N = 6) were taken, unless four distinct regions could not be taken then the entire tissue was imaged. In order to compare surgical to autopsy tissue, as the surgical samples received consisted largely of NP tissue, they were compared with the NP region from autopsy discs; however, it does remain a possibility that neighboring NP tissue (inner AF) could have also been present. Only disc tissue was imaged and quantified excluding granulation tissue. All images were blinded and analyzed using the Nikon Cell Counting software tool within the NIS Elements Advanced Research Software to select for the appropriate color threshold and contrast to identify the percentage of positive cells from the total cells counted.

### Recruitment of mast cells into the IVD

To assess recruitment of mast cells into the IVD, IHC was performed for CCL2/MCP-1 (1:200 Abcam ab9669) and chemoattractant SCF (1:100 Abcam ab64677) on human autopsy and surgical tissue and imaged as described previously.

#### Quantitative real-time Polymerase Chain Reaction (qRT-PCR)

SCF gene expression was also analyzed in human IVD cells from autopsy for each region using qRT-PCR with 1.0 × 10^6^ cells from each group (NP, AF and EP). RNA was extracted by centrifuging the cells at 2,000 × G for 5 minutes, removal of media, and addition of 0.3 mL of Lysis Buffer (Life Technologies) with 1% 2-mercaptoethanol and an equal volume of 70% ethanol. Binding, washing, and elution was carried out using the PuraLink RNA Mini Kit as per manufacturer’s instructions (Life Technologies) and samples converted to cDNA using qScript XLT cDNA SuperMix (Quanta Biosciences). Data was analyzed using the comparative 2 delta delta Ct method, where mRNA levels were normalized to the endogenous control (18 s) and experimental controls^73^.

### The effect of Mast Cell Conditioned Media (MCCM) on IVD Cells

#### Mast Cell Viability in Low Oxygen/Serum Environment

Mast cells were plated at a density of 5.0 × 10^5^ cells/ml in low serum mast cell media (IMDM, 1% FBS, 1% P/S, 1.2 mM α-Thioglycerol) and incubated in 5% O_2_ 5% CO_2_ for 24 hours. Cells were then centrifuged at 0.4 RCF for 5 minutes and incubated in live/dead solution (5 μl Ethidium and 5 μl Calcein/10 ml media) for 5 minutes prior to imaging on the Nikon TiE microscope using a fluorescence DS-Qi2 camera. Images were quantified via the NIS Elements Advanced Research software and assessing the number of live (green) and dead (red) cells.

#### IVD cell viability/proliferation when exposed to MCCM

IVD cells were seeded at a density of 20,000 cells/cm^2^ in 96 well plates and were allowed to equilibrate in basal media for 3 hours. MCCM was generated by culturing mast cells at a concentration of 5.0 × 10^5^ cells/ml with 1 μM calcium ionophore A23187 in low serum media (mast cell basal media) in 5%O_2_, 5%CO_2_ 37 °C for 2.5 hours to induce degranulation^74^. Mast cells were then centrifuged at 0.4 RCF for 5 minutes and freshly isolated MCCM used to treat the IVD cells. Cells were exposed to MCCM for 24 hours at 5%O_2_, 5%CO_2_ 37 °C before being evaluated for cell viability and proliferation. Cell viability was assessed as previously described using calcein/ethidium incubation for 20 minutes^75^. Proliferation was evaluated using 3-(4,5-dimethylthiazol-2-yl)-2,5-diphenyltetrazolium bromide (MTT) assay where 2 mg/ml of 3-(4,5-dimethylthiazol-2-yl)-2,5-diphenyltetrazolium bromide was dissolved in PBS and added to the wells for 1 hour. The cells were then lysed with dimethyl sulfoxide and imaged after 1 minute of incubation at 570 nm (PerkinElmer EnSpire Multimode Plate Reader)^75^.

#### In-vitro Culture Conditions

Bovine IVD cells seeded in 3D agarose gels were examined for their response to mast cell conditioned media (MCCM) (N = 11 NP, N = 11 AF, N = 10 EP). IVD cells were seeded in 350 μl of 2% agarose gel at a final concentration of 4.0 × 10^6^ cells/ml in a 12-well plate^76^ in normal culture conditions (21%O_2_, 5%CO_2_, 37 °C disc cell complete media) for 3 hours. MCCM was gathered as previously described and was added to the gel constructs. Experimental groups included MCCM, 1 μM A23187 in basal media (ionophore control), and basal media. The IVD constructs were then incubated for 24 hours (5%O_2_ 5%CO_2_ 37 °C) after which they were frozen in TRIzol at −80 °C for gene expression analysis using qRT-PCR. mRNA levels of IL-6, CCL2/MCP-1 and ADAMTS5 were assessed as described above for SCF. For samples frozen in TRIzol (IVD agarose constructs), 0.2 mL of chloroform was added per 1 mL of TRIzol, the aqueous upper phase collected, followed by addition of an equal volume of molecular grade 70% Ethanol and use of the Purelink kit described above. Using bovine IVD cells restricted the protein analysis in this portion of the study as there are only a limited number of assays to quantify bovine proteins.

### The effect of Disc Cell Conditioned Media (DCCM) on Mast cells

#### In-vitro Culture Conditions

Mast cells were evaluated for their response to “healthy” and “degenerate” disc cell conditioned media (DCCM). Healthy DCCM was generated by culturing healthy bovine IVD cells in standard disc cell media for 48 hours, while degenerate media was obtained by pre-treating healthy disc cells in disc cell media with 10 ng/ml of TNF-α for 48 hours^77^. TNF-α control, healthy and degenerate DCCM were then applied to 5.0 × 10^5^ mast cells (N = 4 for each group) for 24 hours (5%O_2_ 5%CO_2_ 37 °C). Media was retained and stored at −80 °C to examine mast cell activation/degranulation (Mast Cell Degranulation Kit, EMD Millipore) and VEGFA protein expression. It is important to note that TNF- α has been previously shown to have no effect on degranulation of mast cells^78^. Mast cell lysates were frozen in lysis buffer at −80 °C for qRT-PCR and mRNA gene expression of VEGFA, CCL2, IL-1β, and TNF-α assessed.

#### Mast Cell Activation/Degranulation Assay

Mast cell degranulation was measured using a mast cell degranulation kit (EMD Millipore) to measure the functional amount of mast cell tryptase activity^79^. The assay uses spectrophotometric detection of *p*-nitroaniline (*p*Na) after cleavage from the tryptase substrate tosyl-gly-pro-lys-*p*Na. Media from Healthy and Degenerate DCCM-Mast cell cultures (N = 4) including media from a basal-mast cell control were removed and incubated on 96-well microtiter plate with the tryptase substrate, tosyl-gly-pro-lys-*p*Na, for 1 hour. The plate was then read at a wavelength of 405 nm using a standard microplate reader (PerkinElmer EnSpire Multimode Plate Reader).

#### DCCM-Mast cell interactions on Angiogenesis

To test the angiogenic potential of mast cells in the presence of healthy and degenerate IVD cells, media from the DCCM-Mast cell cultures was evaluated for blood vessel formation using the tubular assay. Human Umbilical Vein Endothelial Cells (HUVECs) were expanded in Medium 200 with Low Serum Growth Supplement (LSGS) (ThermoFisher) and seeded in 96 well plates at 4.0 × 10^4^ cell/well on wells pre-coated with 32 μl of Geltrex (ThermoFisher – A1413201)^64^. Conditions included (1) Standard HUVEC media with LSGS (Positive control), (2) HUVEC media without LSGS (negative control), (3) media from mast cells in basal conditions, and (4) the healthy and (5) degenerate DCCM-mast cell groups (N = 4). Media from each group was filtered using PALL Microsep Advance Centrifugal Devices (PALL Corporation) to retain the soluble factors greater than 3,000 Da and resuspended in HUVEC media (Medium 200) without LSGS before being applied to HUVECs. HUVECs were incubated in conditions described above at 5%CO_2_ at 37 °C for 6 hours. After incubation, 4 mM Calcein AM was added and images captured at ×4 magnification using TiE Nikon Inverted Microscope. The percent (%)area of tubular fluorescence was quantified using ImageJ^80^.

#### DCMM-Mast cell interactions on VEGFA expression

VEGFA protein expression was evaluated in DCCM-Mast cell groups (N = 3) using MesoScale Discovery (MSD: K151RHG-1) Enzyme Linked Immunosorbent Assay (ELISA) for human VEGFA per the manufacturer’s protocol. Briefly, samples were incubated on MSD Human VEGF plate for 2 hours, followed by washing and addition of secondary antibody. The plate was washed again and analyzed on the MESO QuickPlex SQ 120 imager and data analyzed in MSD DISOCVERY WORKBENCH analysis software.

### Statistical Analysis

Due to the small sample size (range of N = 11 to N = 3) an assumption that the data is normally distributed cannot be made and thus non-parametric statistical testing was performed. Non-parametric Mann Whitney tests were used to determine significant differences (p < 0.05) between groups for analysis of mast cell viability (N = 4) and IHC for % of positive Tryptase, SCF and CCL2-MCP-1 cells from surgical (N = 6) and autopsy (N = 7) IVD tissue specimens. A non-parametric Kruskall-Wallis multiple comparison’s test with Dunn’s post hoc was used to determine significant differences (p < 0.05) in gene expression between control and treatment groups for MCCM-IVD cell (N = 10 (EP) and N = 11 (NP and AF) and DCCM-Mast cell (N = 4) studies including the Mast cell Degranulation assay (N = 4), VEGFA protein expression (N = 3) and tubular/angiogenesis assay (N = 4).

## Electronic supplementary material

Dataset 1
