# Supplementary material for: Mast Cell-Intervertebral disc cell interactions regulate inflammation, catabolism and angiogenesis in Discogenic Back Pain
Source: Sci Rep. 2017 Oct 2;7:12492. doi: 10.1038/s41598-017-12666-z (PMC5624870; doi:10.1038/s41598-017-12666-z)

**Mast Cell-Intervertebral disc cell interactions regulate inflammation, catabolism and angiogenesis in Discogenic Back Pain**

Authors: Matthew G. Wiet BS^1^; Andrew Piscioneri^1^; Safdar N. Khan MD^2^; Megan N. Ballinger PhD^3^; Judith A. Hoyland PhD^4, 5^; Devina Purmessur PhD^1, 2^*

^1^Department of Biomedical Engineering, The Ohio State University, Columbus Ohio, 201 Davis Heart and Lung Research Institute, 473 W 12th Avenue, Columbus, Ohio 43210

^2^Department of Orthopedics, The Ohio State University Wexner Medical Center, 1070 OSU CarePoint East, 543 Taylor Avenue, Columbus, Ohio 43203

^3^Department of Internal Medicine, Division of Pulmonary, Critical Care and Sleep Medicine, The Ohio State University, 201 Davis Heart and Lung Research Institute, 473 West 12^th^ Avenue, Columbus, Ohio 43210

^4^Division of Cell Matrix Biology and Regenerative Medicine, School of Biological Sciences, Faculty of Biology, Medicine and Health, The University of Manchester, Stopford Building, Oxford Road, Manchester, M13 9PT, United Kingdom

^5^ NIHR Manchester Musculoskeletal Biomedical Research Centre, Manchester Academic Health Science Centre, Central Manchester NHS Foundation Trust, Manchester, United Kingdom

*Corresponding Author: Devina Purmessur (Correspondence to [walter.368@osu.edu](mailto:walter.368@osu.edu); devina.purmessur@osumc.edu)

**Supplemental Dataset 1**

Supplemental Figure 1: (A) Ethidium/Calcein stain of mast cells in normal mast cell environment, as well as in IVD environment. (B) Mast cells maintain similar levels of viability in both normal and low oxygen/serum environments (p>0.05).

Supplemental Figure 2: SCF qRT-PCR gene expression in human IVD cells. Ct values relative to 18s for each region of the IVD (NP, AF, EP).

Supplemental Figure 3: Calcein/Ethidium staining for cell viability and MTT proliferation absorbance measurements for IVD cells exposed to MCCM for 24 hour period. There was not a significant difference in viability or proliferation of cells exposed to MCCM (p>0.05).

Supplemental Figure 4: Positive and negative controls for tubular formation assay.


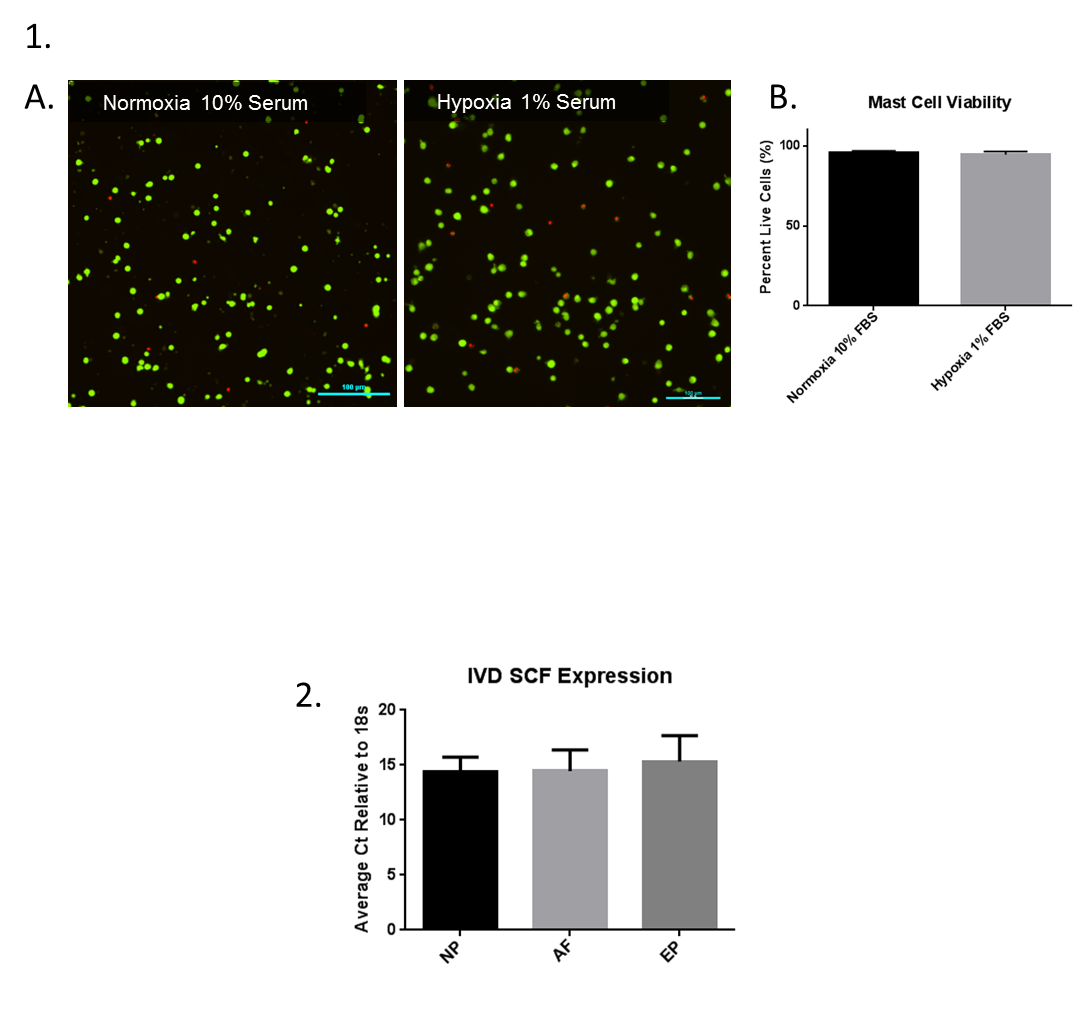


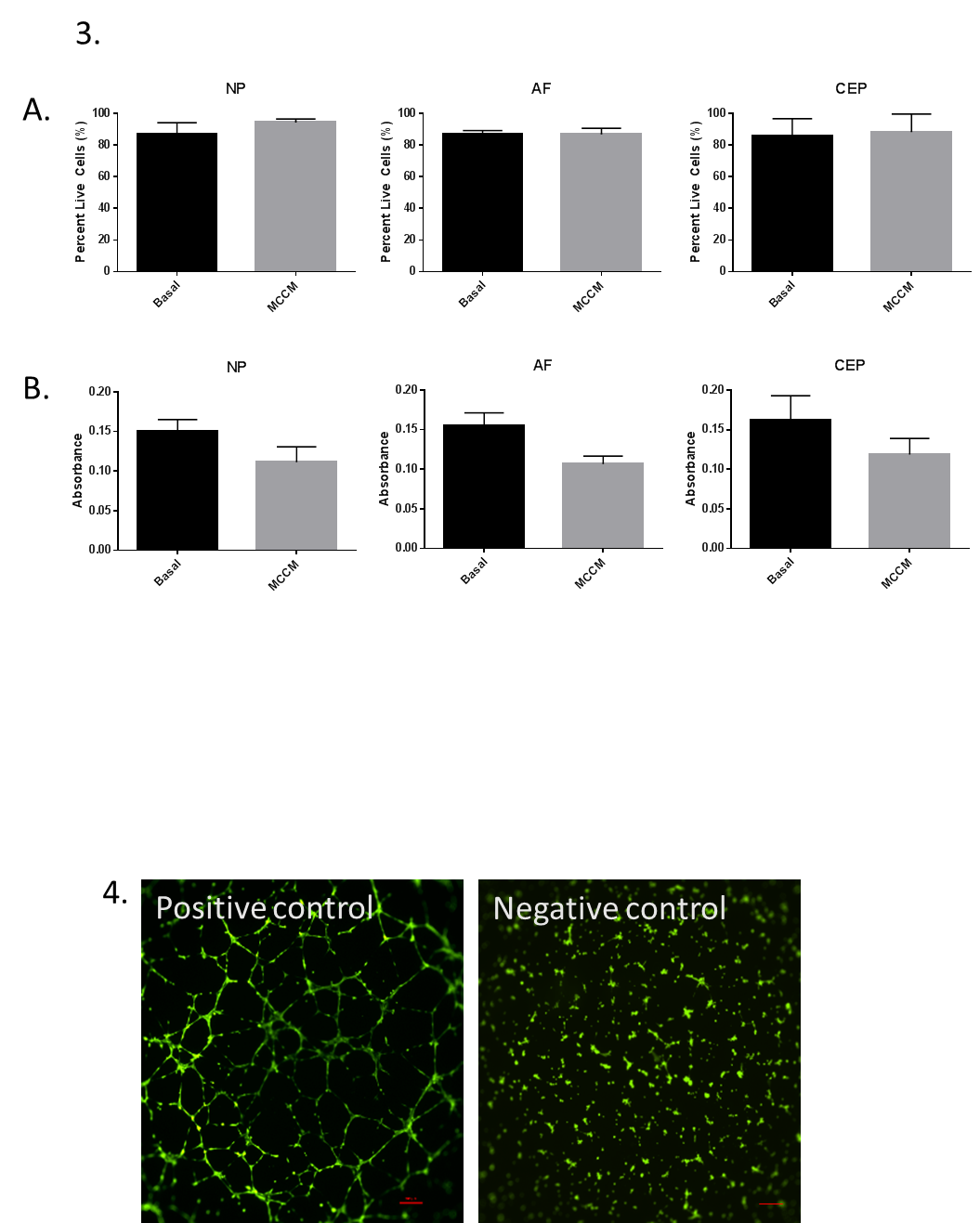

Supplement: Supplementary file 1 — Dataset 1 [file 41598_2017_12666_MOESM1_ESM.doc]
